# The P2Y_2_ receptor mediates terminal adipocyte differentiation and insulin resistance: Evidence for a dual G-protein coupling mode

**DOI:** 10.1016/j.jbc.2023.105589

**Published:** 2023-12-21

**Authors:** Shenqi Qian, Yi Shi, Jared Senfeld, Qianman Peng, Jianzhong Shen

**Affiliations:** 1 Department of Drug Discovery and Development, Harrison College of Pharmacy, Auburn University, Auburn, Alabama, USA; 2 Department of Pharmacy, Second Affiliated Hospital, Zhejiang University School of Medicine, Hangzhou, China

**Keywords:** P2Y_2_ receptor, obesity, insulin resistance, AKT, adipocytes

## Abstract

Several P2Y nucleotide receptors have been shown to be involved in the early stage of adipocyte differentiation *in vitro* and insulin resistance in obese mice; however, the exact receptor subtype(s) and its underlying molecular mechanism in relevant human cells are unclear. Here, using human primary visceral preadipocytes as a model, we found that during preadipocyte-to-mature adipocyte differentiation, the P2Y_2_ nucleotide receptor (P2Y_2_R) was the most upregulated subtype among the eight known P2Y receptors and the only one further dramatically upregulated after inflammatory TNFα treatment. Functional studies indicated that the P2Y_2_R induced intracellular Ca^2+^, ERK1/2, and JNK signaling but not the p38 pathway. In addition, stimulation of the P2Y_2_R suppressed basal and insulin-induced phosphorylation of AKT, accompanied by decreased GLUT4 membrane translocation and glucose uptake in mature adipocytes, suggesting a role of P2Y_2_R in insulin resistance. Mechanistically, we found that activation of P2Y_2_R did not increase lipolysis but suppressed PIP_3_ generation. Interestingly, activation of P2Y_2_R triggered G_i_-protein coupling, and pertussis toxin pretreatment largely inhibited P2Y_2_R-mediated ERK1/2 signaling and cAMP suppression. Further, treatment of the cells with AR-C 118925XX, a selective P2Y_2_R antagonist, significantly inhibited adipogenesis, and P2Y_2_R knockout decreased mouse body weight gain with smaller eWAT mass infiltrated with fewer macrophages as compared to WT mice in response to a Western diet. Thus, we revealed that terminal adipocyte differentiation and inflammation selectively upregulate P2Y_2_R expression and that P2Y_2_R mediates insulin resistance by suppressing the AKT signaling pathway, highlighting P2Y_2_R as a potential new drug target to combat obesity and type-2 diabetes.

Obesity is a public health problem with increasing worldwide prevalence and severe complications over time, such as type-2 diabetes mellitus, nonalcoholic fatty liver disease, metabolic syndrome, chronic kidney disease, cardiovascular disease, hyperlipidemia, depression, and some cancers (1, 2, 3, 4). Severe obesity (body mass index >35) is associated with about a 4-fold increased risk of incident heart failure and a 2-fold increased risk of incident coronary heart disease and stroke (5, 6). Recent research shows that obesity will decrease life expectancy by 7 years at the age of 40 years (7). Obesity also imposes a significant economic burden on the patients themselves and their families and nations (8, 9). Most people believe the fundamental cause of fat accumulation is an energy imbalance between calorie intake and consumption. The recommended therapy includes lifestyle intervention, pharmacotherapy, and surgery (https://www.niddk.nih.gov/health-information/weight-management/adult-overweight-obesity/treatment). Pharmacotherapy should be a consideration for patients with comorbid conditions who failed to achieve weight loss from lifestyle modification and with difficulty maintaining long-term weight loss. Current drugs used for the prevention or treatment of obesity lack efficacy and consistent effect or have been withdrawn from the market by the FDA due to severe side effects (https://www.niddk.nih.gov/health-information/weight-management/adult-overweight-obesity/treatment), (10). Thus, there is an urgent need to identify new therapeutic targets to control or treat obesity.

Obesity is associated with low-grade inflammation and insulin resistance. The insulin receptors bind with insulin, a peptide hormone secreted by the β-cells of the pancreas, to regulate glycogen deposition, stimulate lipogenesis, inhibit lipolysis, and increase glucose uptake (11, 12, 13). Evidence shows that dysfunction of insulin receptor downstream PI3K/AKT signaling pathway leads to the development of obesity and type-2 diabetes mellitus because AKT activation promotes glucose transporter 4 (GLUT4) translocation from cytosol to the cell membrane and uptake of excess glucose in the bloodstream (14, 15, 16). Studies also found that chronic inflammation mediated by macrophages and other immune cells in adipose tissues contributes to the development of insulin resistance (17). The current concept is that macrophages release pro-inflammatory mediators, including TNFα, to activate the JNK signaling pathway, leading to insulin receptor desensitization by JNK phosphorylation of the insulin receptor substrates (18). However, this working model cannot fully explain why some of the post-insulin receptor signaling pathways, such as ERK1/2, are not compromised as much as the AKT pathway, suggesting that there is an additional unknown mechanism(s) that can selectively impair the insulin receptor-induced AKT pathway, leading to less GLUT4-mediated glucose uptake in relevant organs or tissues including white adipocytes that play a pivotal role in glucose homeostasis and obesity.

Purinergic signaling has been investigated as a therapeutic strategy for obesity and diabetes recently. Using adenosine A1 receptor–deficient mice, it was shown that activation of this receptor in rodents has anti-lipolytic effects mediated by the inhibition of intracellular cAMP production and a decrease in PKA and lipase activities (19, 20, 21). In contrast, mice overexpressing the A1 receptor in adipose tissues are protected from obesity-induced insulin resistance (22). In addition, P2Y_4_ receptor activation inhibited cardiac adipose tissue–derived stem cell differentiation, and mice with P2Y_4_ receptor deletion developed more cardiac adipose tissue mass (23). Interestingly, the P2X7 receptor–deficient mice had increased body weight, adipocyte hyperplasia in fat pads, and ectopic lipid accumulation in the kidney, salivary glands, and pancreas (24).

P2Y_2_ receptor (P2Y_2_R) is a G protein–coupled purinergic receptor that selectively responds to extracellular ATP and UTP. It is expressed in all insulin-sensitive and metabolically essential tissues, including adipose tissues, the liver, and the skeletal muscles. Using a global P2Y_2_R KO mouse model, it was reported that P2Y_2_R plays a significant role in diet-induced obesity and facilitates high-fat diet-induced insulin resistance (25). However, the exact role of P2Y_2_R in adipocytes or other tissues remains unknown. Another independent study indicates that the P2Y_2_R expressed in myeloid cells is not responsible for high-fat diet–induced systemic inflammation, insulin resistance, and obesity (26). These led us to hypothesize that inflammation promotes adipocyte P2Y_2_R upregulation, which leads to insulin resistance by inhibiting the insulin receptor-AKT signaling pathway. Thus, the principal objective of this study is to determine whether terminal human adipocyte differentiation, along with inflammation, promotes P2Y_2_R upregulation and to explore the new mechanism(s) underlying P2Y_2_R control of insulin resistance.

## Results

### P2Y_2_R upregulation during terminal adipocyte differentiation and further selectively increased after inflammation

**Figure 1:**
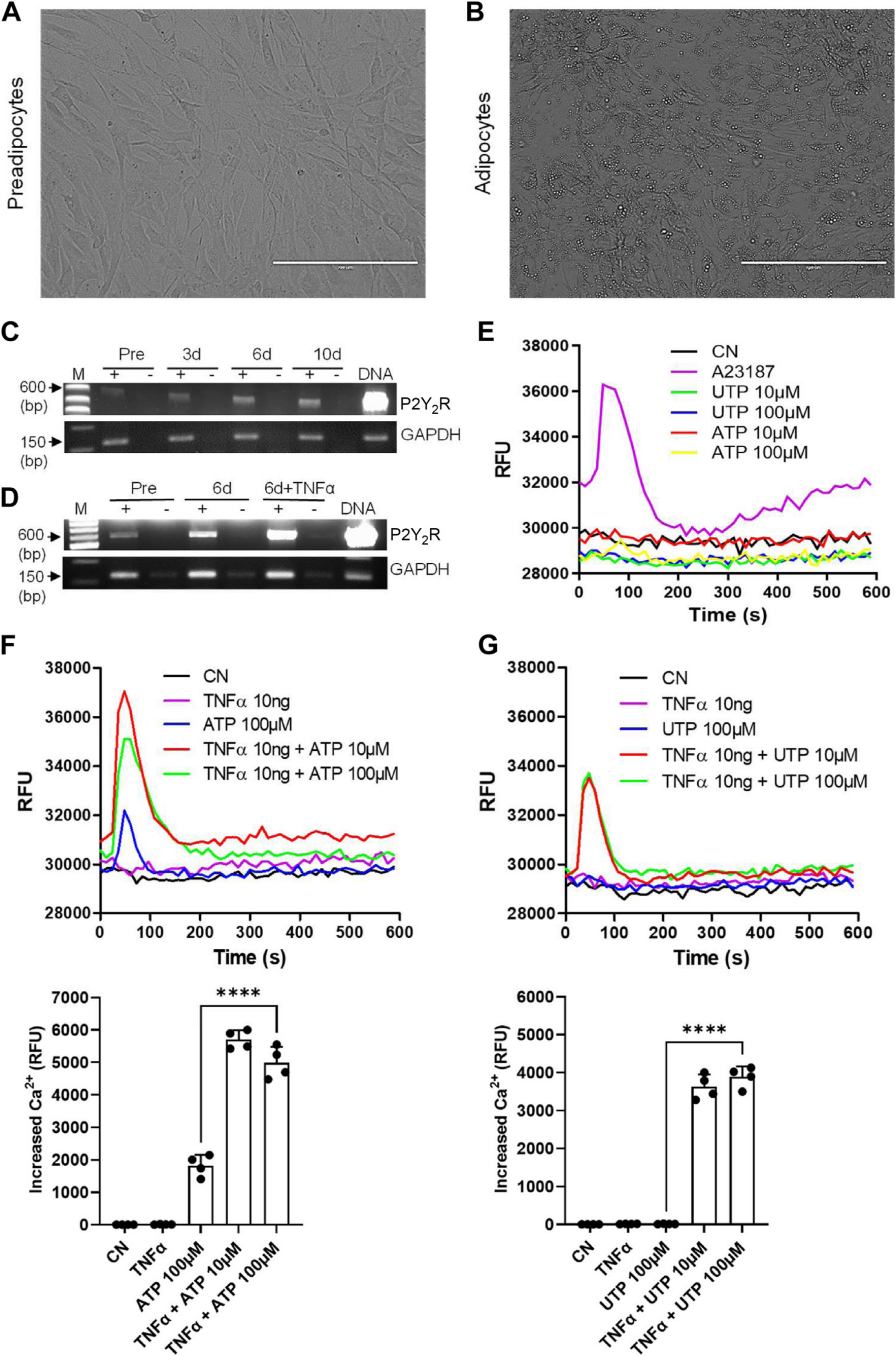
**P2Y_2_R upregulation during adipogenesis and inflammation.***A* and *B*, representative cell morphology of human visceral preadipocytes and differentiated mature adipocytes. Bar scale represents 200 μm. *C*, P2Y_2_R mRNA was upregulated during terminal adipocyte differentiation from 3 to 10 days. Representative data from three independent experiments. *D*, stimulation of the mature adipocytes by TNFα (10 ng/ml) for 24 h further increased P2Y_2_R mRNA expression. The GAPDH is used as a loading control. Representative data from three independent experiments. P2Y_2_R agonist ATP- or UTP-induced intracellular Ca^2+^ increase was determined in preadipocytes (*E*) and mature adipocytes with or without TNFα (10 ng/ml) pretreatment for 24 h (*F* and *G*). A23187 (10 μM) served as a positive control that induced Ca^2+^ mobilization (*E*). Shown are representatives of four independent experiments with summarized data presented in the bottom figures. ∗∗∗∗*p* < 0.0001. P2Y_2_R, P2Y_2_ receptor.

To explore a possible expression change of P2Y_2_R during preadipocyte-to-mature adipocyte differentiation, we cultured human primary visceral preadipocytes in a defined differentiation media for different days during which total cellular RNAs were isolated from the cells differentiated for 0, 3, 6, and 10 days Figure 1, *A* and *B* shows representative cell morphology of preadipocytes before differentiation, and the mature adipocytes differentiated for 6 days with expected lipid droplet formation. This adipocyte differentiation was accompanied by the upregulation of several adipogenic markers, including C/EBPβ, C/EBPα, and PPARγ (Fig. S1). Real-time RT-PCR analysis showed that P2Y_2_R mRNA expression was upregulated during adipocyte terminal differentiation and reached the maximum at day 6 (Fig. 1*C*). To determine whether inflammation affects the expression of P2Y_2_R mRNA, the mature adipocytes were treated with TNFα (10 ng/ml, 24 h) to induce inflammation. RT-PCR analysis showed that P2Y_2_R mRNA expression was further increased dramatically after TNFα stimulation compared with control cells without TNFα (Fig. 1*D*). Interestingly, although we found multiple P2Y receptors including P2Y_2_, P2Y_12_, and P2Y_13_ were significantly upregulated during the terminal differentiation, only the P2Y_2_R in mature adipocytes was further upregulated by TNFα challenge (Fig. S2, *A* and *B*). Real-time RT-PCR analysis further confirmed this phenomenon in primary human adipocytes and mouse 3T3-L1 adipocytes (Fig. S2, *C* and *D*). These data indicate that P2Y_2_R mRNA was upregulated during terminal adipogenesis and further selectively upregulated by inflammation.

To assess whether the P2Y_2_R mRNA was translated into a functional protein during adipocyte differentiation, a Ca^2+^ mobilization assay was performed since the P2Y_2_R is linked to the release of intracellular Ca^2+^ through Gq proteins. P2Y_2_R agonist ATP and UTP were used to induce intracellular Ca^2+^ mobilization in preadipocytes and mature adipocytes. There was no Ca^2+^ signal detectable on preadipocytes after ATP or UTP treatment up to 100 μM, but 10 μM A23187, as a positive control, induced expected Ca^2+^ signaling (Fig. 1*E*), which is consistent with the fact that there is a barely detectable level of P2Y_2_R mRNA in preadipocytes. However, after complete differentiation, UTP (10–100 μM) stimulation still did not induce any noticeable Ca^2+^ signal in these mature adipocytes, with a small response after ATP (100 μM) stimulation (Fig. 1, *F* and *G*). Interestingly, after the induction of inflammation (TNFα 10 ng/ml, 24 h pretreatment followed by 3-h washout), both ATP and UTP stimulation induced robust Ca^2+^ signal (Fig. 1, *F* and *G*). Collectively, these results indicate that inflammation triggered not only P2Y_2_R mRNA upregulation but also the upregulation of the functional P2Y_2_R proteins.

### P2Y_2_R downstream signaling in preadipocytes *versus* mature adipocytes with and without inflammation

**Figure 2:**
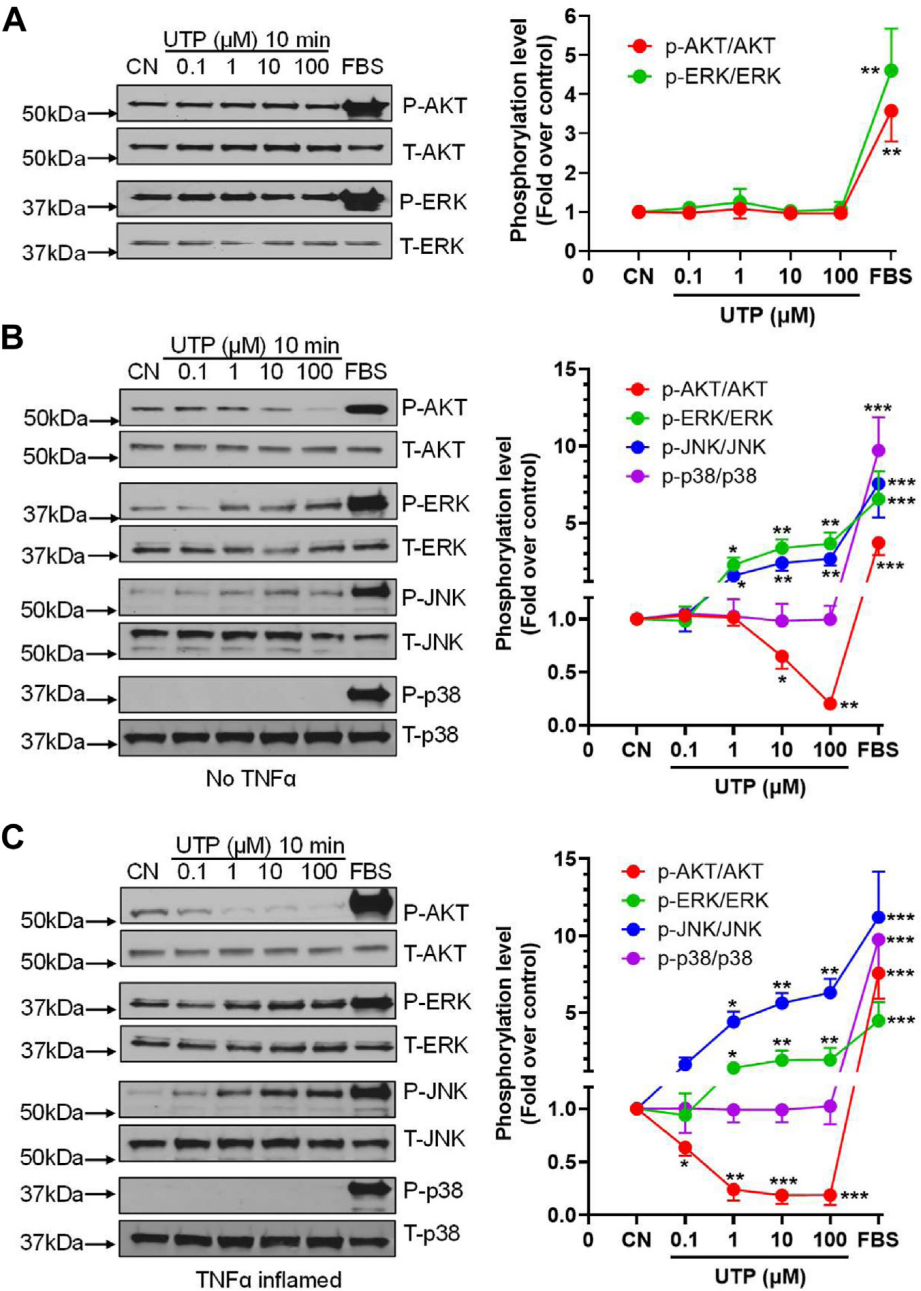
**P2Y_2_R signaling profiles in preadipocytes *versus* mature adipocytes with and without inflammation.** Phosphorylation of AKT, ERK1/2, JNK, and p38 was determined by Western blotting assays. *A*, stimulation of the P2Y_2_R by UTP (0.1–100 μM) for 10 min showed no effect on phosphorylation of AKT and ERK1/2 in preadipocytes. *B*, in contrast, UTP suppressed the phosphorylation of AKT and stimulated the phosphorylation of ERK1/2 and JNK in a dose-dependent manner with no effect on the phosphorylation of p38 in mature adipocytes. *C*, inflammation induction by TNFα (10 ng/ml pretreatment for 24 h) further promoted UTP-induced effects. FBS (5%) was used as a positive control. Shown are representatives of three independent experiments. All figures on the right side represent the summarized data respectively. ∗*p* < 0.05; ∗∗*p* < 0.01; ∗∗∗*p* < 0.001. P2Y_2_R, P2Y_2_ receptor.

To further determine the P2Y_2_R downstream signaling, we performed a dose-response study using UTP because it is more selective than ATP towards the P2Y_2_R in our system. Figure 2*A* shows that UTP up to 100 μM did not activate the ERK1/2 and AKT pathways in preadipocytes, consistent with what we observed in our Ca^2+^ mobilization assay. However, to our surprise, UTP stimulation in healthy mature adipocytes inhibited AKT phosphorylation while stimulating phosphorylation of ERK1/2 and JNK pathways in a dose-dependent manner, with no effect on the p38 pathway (Fig. 2*B*). Notably, after TNFα treatment to induce inflammation on these mature adipocytes, UTP induced a much more robust inhibition on AKT phosphorylation with an increased potency as well. In addition, the ERK1/2 and JNK, but not the p38 pathways, were also more dramatically activated by UTP in these inflamed mature adipocytes (Fig. 2*C*).

### Validation of P2Y_2_R involvement *via* pharmacological antagonism and siRNA silencing

**Figure 3:**
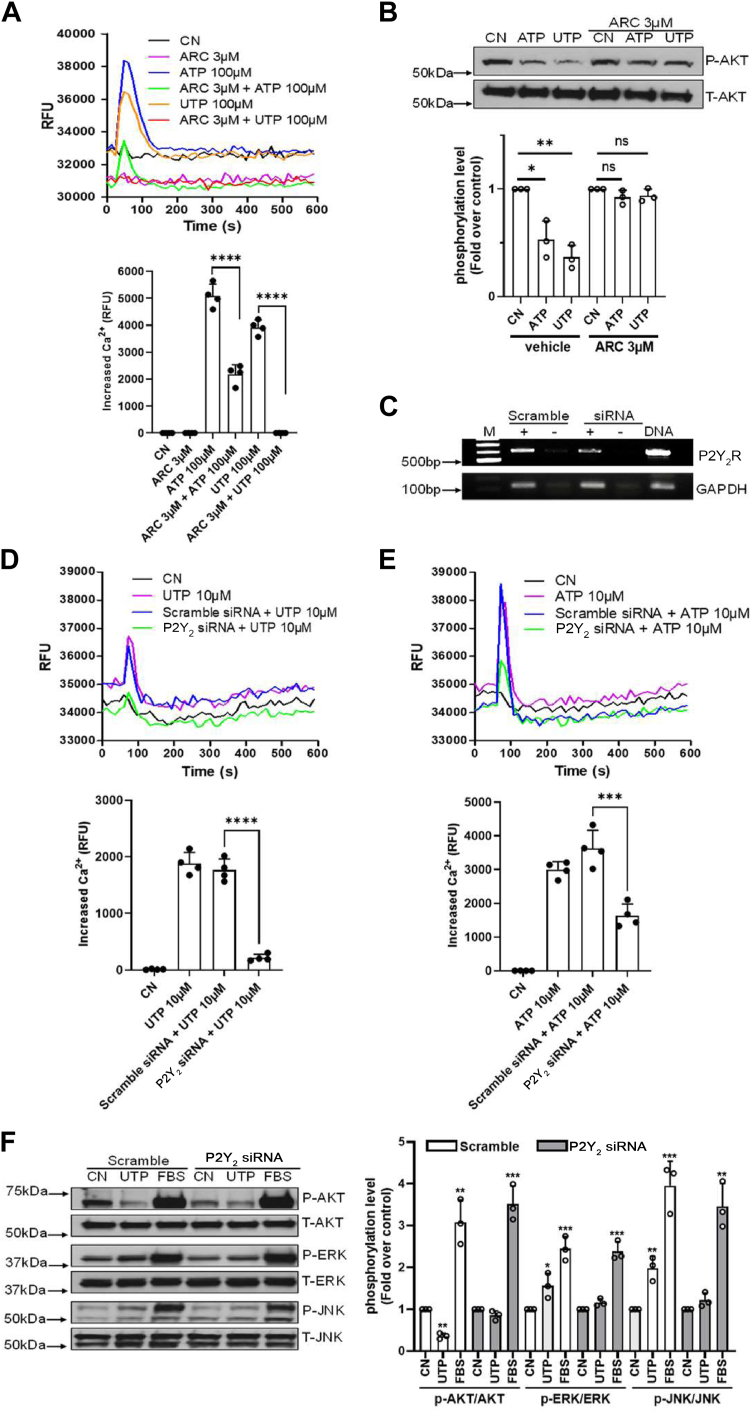
**Validation of functional P2Y_2_R involvement *via* pharmacological antagonism and siRNA silencing.***A*, AR-C118925 (ARC, 3 μM) pretreatment for 45 min significantly suppressed or eliminated ATP/UTP-induced (100 μM) intracellular Ca^2+^ mobilization. Representative tracings are shown on the *top* and summarized data shown on the *bottom*. ∗∗∗∗*p* < 0.0001. *B*, ATP/UTP-induced AKT pathway inhibition was prevented by pretreatment of the cells with AR-C118925 (ARC, 3 μM for 45 min). Summarized data shown on the *bottom*. ∗*p* < 0.05; ∗∗*p* < 0.01. *C*, the level of P2Y_2_R mRNA dramatically decreased after treating the cells with P2Y_2_R-selective siRNA (25 nM) compared to scramble control siRNA (25 nM) as determined by RT-PCR. Scramble control siRNA was used as a negative control *D* and *E*, ATP- or UTP-induced (10 μM) intracellular Ca^2+^ increase was dramatically reduced after P2Y_2_R siRNA silencing compared to scramble control siRNA. Representative tracings are shown on the *top* and summarized data shown on the *bottom*. ∗∗∗*p* < 0.001; ∗∗∗∗*p* < 0.0001. *F*, Western blotting assays showed that UTP-induced inhibition of AKT phosphorylation and activation of ERK1/2 and JNK phosphorylation were all reduced by P2Y_2_R siRNA silencing compared to scramble control siRNA. Shown are representatives of three independent experiments with summarized data shown on the right. ∗∗*p* < 0.01; ∗∗∗*p* < 0.001. P2Y_2_R, P2Y_2_ receptor.

To validate whether P2Y_2_R genuinely mediated our observed results, we employed the P2Y_2_R-selective antagonist AR-C118925 (3 μM for 45 min pretreatment) before ATP/UTP stimulation on mature adipocytes. We found that ATP-induced (100 μM) Ca^2+^ mobilization was dramatically reduced, whereas UTP-induced (100 μM) Ca^2+^ mobilization was abolished (Fig. 3*A*), suggesting that the P2Y_2_R mediates the UTP response and that there was a minor contribution of another receptor for the ATP response beyond the P2Y_2_R. Next, we evaluated whether P2Y_2_R mediates the inhibitory effect on AKT. Figure 3*B* shows that inhibition of AKT phosphorylation by ATP/UTP (100 μM) was diminished when the cells were pretreated with AR-C 118925, indicative of a role of the P2Y_2_R.

To further confirm the involvement of P2Y_2_R, we transfected mature adipocytes with P2Y_2_R siRNA and the scrambled control siRNA. Real-time RT-PCR analysis showed that P2Y_2_R siRNA treatment significantly reduced P2Y_2_R mRNA expression compared with scrambled control siRNA treatment (Fig. 3*C*). The Ca^2+^ mobilization assay indicated that mature adipocytes transfected with scrambled control siRNA remained at a similar level of Ca^2+^ mobilization as in non-siRNA–treated control cells in response to UTP/ATP stimulation, but P2Y_2_R siRNA transfection dramatically reduced the Ca^2+^ mobilization induced by ATP/UTP (Fig. 3, *D* and *E*), supporting a role for the P2Y_2_R. Western blotting assays further showed that a similar pattern of UTP-induced inhibition of AKT phosphorylation and activation of ERK1/2 and JNK phosphorylation were obtained by scrambled control siRNA, but these effects were reduced by P2Y_2_R siRNA treatment (Fig. 3*F*). Interestingly, whenever the mature adipocytes were treated with AR-C118925 or the P2Y_2_R-selective siRNAs, we consistently observed a lower basal level of intracellular Ca^2+^ signal (Fig. 3, *A*, *D* and *E*).

### P2Y_2_R activation selectively blocks insulin-induced AKT signaling in mature adipocytes

**Figure 4:**
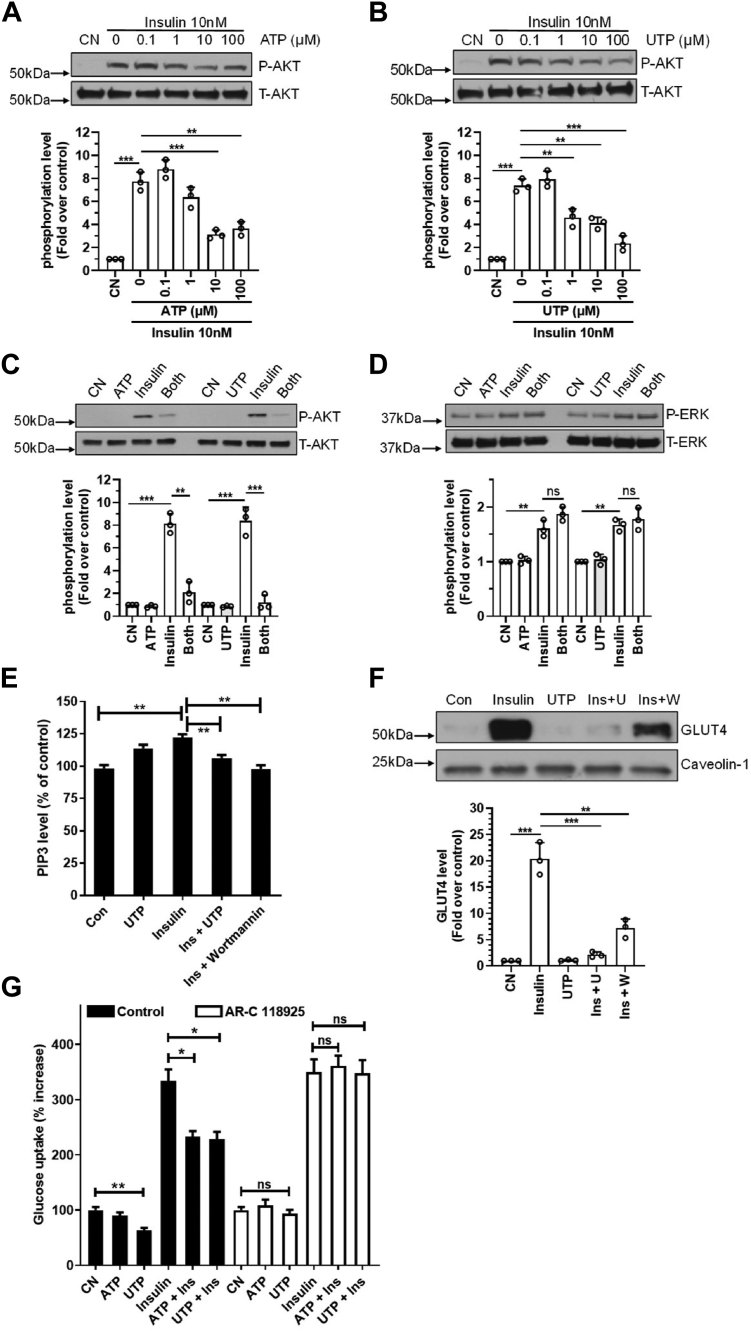
**P2Y_2_R activation selectively compromises insulin-induced AKT activation and glucose uptake in mature adipocytes.***A* and *B*, ATP or UTP dose-dependently inhibited insulin-induced AKT phosphorylation. The figures on the *bottom* represent the summarized data, respectively. ∗∗*p* < 0.01; ∗∗∗*p* < 0.001. *C* and *D*, hundred micromolars of ATP or UTP alone did not induce any AKT phosphorylation, but they blocked 10 nM insulin-induced AKT but not EKR1/2 phosphorylations in mature adipocytes. The figures on the *bottom* represent the summarized data respectively. ∗∗*p* < 0.01; ∗∗∗*p* < 0.001. *E*, P2Y_2_R activation by 100 μM UTP suppressed 10 nM insulin-induced PIP3 generation determined by ELISA. Hundred nanomolars of Wortmannin was used as a positive control. ∗∗*p* < 0.01, n = 7. *F*, ten nanomolars of insulin stimulation of the cells led to a dramatic increase of membrane GLUT4 detected by Western blotting assays, which was largely suppressed by 100 μM UTP cotreatment. Hundred nanomolars of Wortmannin was used as a positive control. Caveolin-1 served as a protein loading control. Shown are representatives of three independent experiments with summarized data shown on the *bottom*. ∗∗*p* < 0.01; ∗∗∗*p* < 0.001. *G*, ten nanomolars of insulin-induced glucose uptake was significantly inhibited by cotreatment of the cells with either 100 μM ATP or UTP. AR-C118925 (ARC, 3 μM) pretreatment for 45 min abolished the suppressive effects of ATP and UTP. ∗*p* < 0.05, ∗∗*p* < 0.01, n = 9. GLUT4, glucose transporter 4; P2Y_2_R, P2Y_2_ receptor.

Because we found that activation of the P2Y_2_R inhibited the basal cellular level of AKT phosphorylation (Fig. 2), we further explored whether insulin-induced AKT activation can be affected by the P2Y_2_R. Figure 4, *A* and *B* show that ATP and UTP dose-dependently inhibited insulin-induced AKT phosphorylation, with an apparent IC50 around 10 μM. Interestingly, cotreatment of the cells with even a maximal dose of ATP or UTP at 100 μM together with 10 nM insulin for 10 min did not affect insulin-induced ERK1/2 phosphorylation, but the same treatment led to a consistent inhibition of AKT phosphorylation (Fig. 4, *C* and *D*). These data indicate that activation of the P2Y_2_R induces a biased inhibition of insulin receptor signaling towards the AKT pathway, a phenomenon well-known for insulin resistance.

To further explore a possible mechanism underlying P2Y_2_R inhibition of insulin-AKT signaling, we determined whether P2Y_2_R affects PI3K-mediated PIP3 production by performing the in-cell PIP3 Elisa Assay. Figure 4*E* shows that stimulation of the cells with 10 nM insulin induced a significant increase of PIP3 as expected; however, cotreatment with UTP significantly blocked insulin-induced PIP3 generation as much as the PI3K inhibitor Wortmannin did.

### P2Y_2_R suppresses insulin-induced GLUT4 membrane translocation and glucose uptake in mature adipocytes

Next, we evaluated whether P2Y_2_R inhibition of the insulin–AKT signaling axis impacts glucose transport. Figure 4*F* shows that insulin stimulation increased membrane GLUT4 protein content dramatically compared with control; however, cotreatment with UTP largely inhibited insulin-induced GLUT4 membrane translocation, an effect seemingly more efficacious than Wortmannin. Of note, the same treatment did not affect the levels of total cellular GLUT4 protein expression (Fig. S3). Consistent with this result, we found that activation of the P2Y_2_R with either ATP or UTP all significantly inhibited insulin-induced glucose uptake, though UTP stimulation alone decreased basal glucose uptake as well. In addition, this suppressive effect of ATP/UTP was totally blocked by pretreatment of the cells with AR-C 118925 (Fig. 4*G*). Collectively, these data clearly suggested that P2Y_2_R contributes to insulin resistance in adipocytes.

### P2Y_2_R activation leads to the inhibition of lipolysis

**Figure 5:**
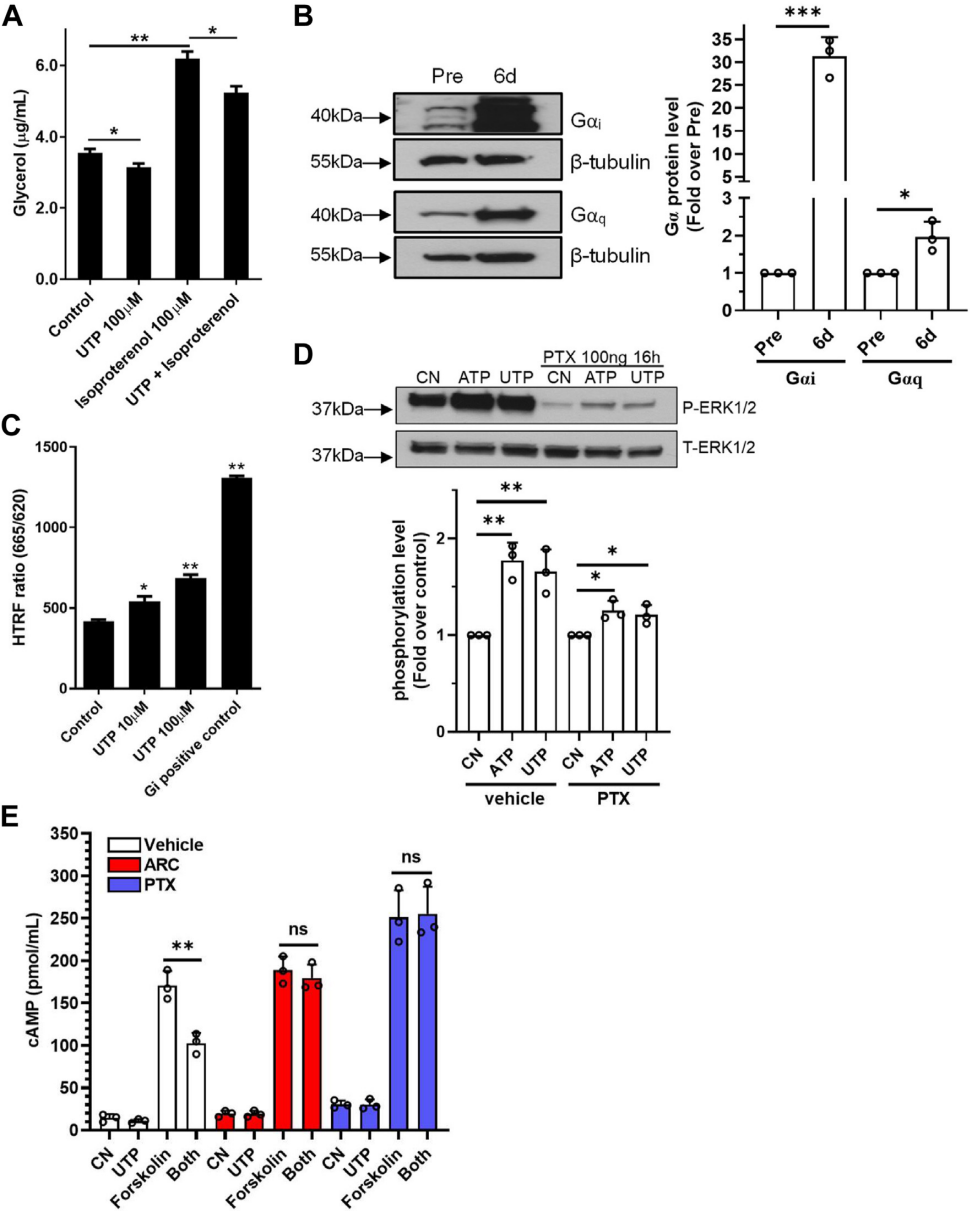
**Evidence of Gi protein coupling and inhibition of lipolysis after P2Y_2_R activation in mature adipocytes.***A*, UTP stimulation of mature adipocytes decreased glycerol release during adipolysis, while isoproterenol stimulation, as a positive control, dramatically increased glycerol release, which was decreased by UTP cotreatment. ∗*p* < 0.05; ∗∗*p* < 0.01, n = 6. *B*, dramatic upregulation of Gαi proteins and a mild induction of Gαq proteins after the human visceral preadipocyte differentiation for 6 days. Shown are representatives of three independent experiments with summarized data shown on the *right*. ∗*p* < 0.05; ∗∗∗*p* < 0.001. *C*, Gi protein binding assay by HTRF showed that stimulation of P2Y_2_R by UTP dose dependently increased the activation of Gi proteins. ∗*p* < 0.05; ∗∗*p* < 0.01, n = 7. *D*, Gi protein inhibitor Pertussis toxin (PTX, 100 ng/ml) pretreatment for 16 h dramatically reduced ATP- and UTP-induced phosphorylation of ERK1/2 determined by Western blotting assays. Shown are representatives of three independent experiments with summarized data shown on the *bottom*. ∗*p* < 0.05; ∗∗*p* < 0.01. *E*, stimulation of P2Y_2_R by UTP (100 μM) resulted in an inhibition of forskolin (10 μM)-induced cAMP elevation in mature adipocytes. Pretreatment with AR-C118925 (ARC, 3 μM) for 45 min or with PTX (100 ng/ml) for 16 h all abolished the inhibitory effect of UTP. cAMP assays were performed by ELISA as described in Experimental procedures. Shown are the summarized data from three independent experiments performed in triplicates. ∗∗*p* < 0.01. P2Y_2_R, P2Y_2_ receptor.

Since increased lipolysis was known to be a causal effect on insulin resistance, we evaluated whether P2Y_2_R activation affects lipolysis. Figure 5 shows that stimulation of the P2Y_2_R with UTP slightly but significantly decreased the cellular release of glycerol into the medium, whereas isoproterenol, a positive control, dramatically increased glycerol release which was significantly reduced by UTP cotreatment. This indicates that P2Y_2_R-induced insulin resistance is unlikely due to free fatty acids. A recent study reported that activation of the adipocyte P2Y_2_R decreases cellular cAMP levels, but the mechanism was not clearly documented (27). We hypothesized that the P2Y_2_R might be coupled with Gi proteins in these mature human adipocytes, which could explain why it can decrease cellular cAMP and suppress lipolysis. To test this idea, we first checked the Gi protein expression during terminal differentiation. Figure 5*B* shows that although both Gαq and Gαi were upregulated in fully differentiated cells compared with the preadipocytes, the Gαi upregulation was much more overwhelming. We then performed a Gi protein-binding assay. Figure 5*C* shows that stimulation of the mature adipocyte P2Y_2_R with UTP dose dependently increased GTP binding to the Gαi proteins, indicating a Gi protein coupling after P2Y_2_R activation. Consistent with this, pretreatment of the cells with pertussis toxin (PTX), a specific inhibitor of Gi proteins, significantly blocked ATP- and UTP-induced signaling to the ERK1/2 pathway (Fig. 5*D*). In addition, we found that activation of the P2Y_2_R by UTP significantly suppressed forskolin-induced cAMP elevation, which was entirely prevented by pretreatment of the cells with either AR-C 118925 or PTX (Fig. 5*E*).

### Role of P2Y_2_R in adipogenesis *in vitro* and *in vivo*

**Figure 6:**
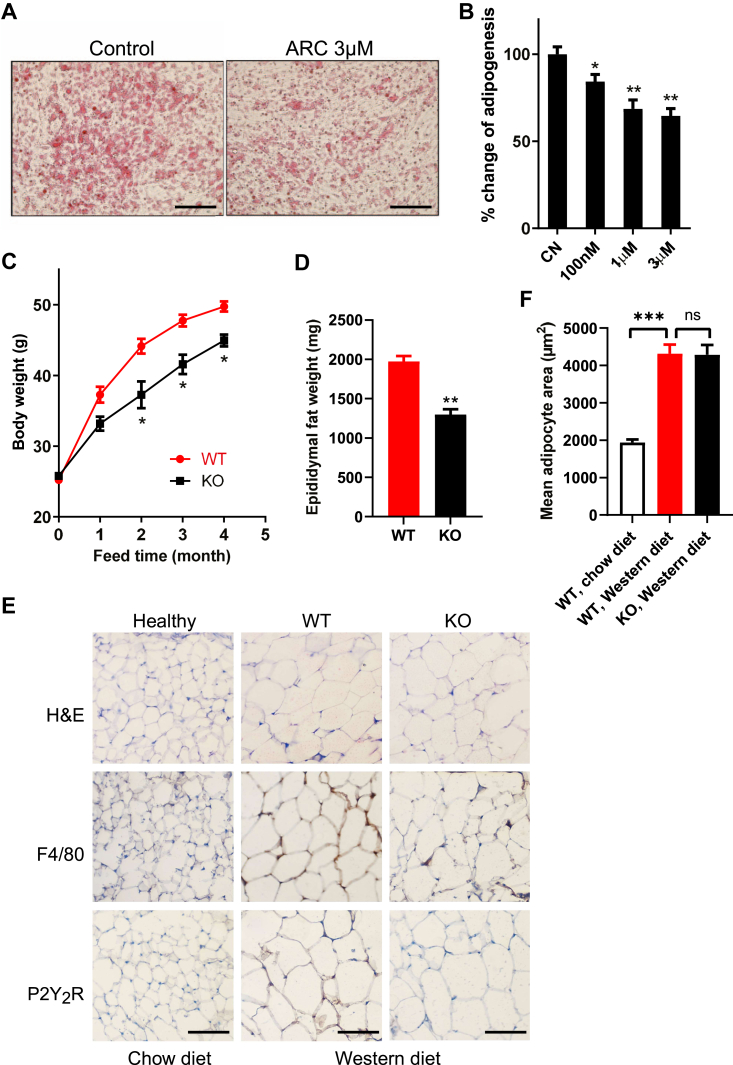
**Role of P2Y_2_R in adipogenesis *in vitro* and *in vivo*.***A*, human visceral preadipocytes were differentiated for 6 days in the absence (control) or presence of different doses of AR-C 118925 (ARC), after which lipid droplet formation during adipogenesis was stained by Oil Red-O and then extracted for quantifications. Shown are representative images of five independent experiments (*A*), with quantified data shown in (*B*). Bar scale represents 200 μm. ∗*p* < 0.05, ∗∗*p* < 0.01, n = 5 (*B*). *C* and *D*, changes of body weight and epididymal fat weight over the entire experiment from 2-month-old male WT and P2Y_2_R-KO mice fed a Western diet for 4 months ∗*p* < 0.05, ∗∗*p* < 0.01, n = 7 in each group. *E*, H&E, F4/80 (macrophages), and P2Y_2_R staining were applied to detect *in vivo* expression of macrophages and P2Y_2_R in mouse epididymal fat tissue sections isolated from WT and KO mice fed with a Western diet for 4 months. WT mice fed a normal chow diet were served as healthy control. Scale bar represents 200 μm. *F*, histological H&E-stained slides of mouse epididymal fat tissue sections were used to calculate mean adipocyte areas. ∗∗∗*p* < 0.001, n = 7 mice in each group. P2Y_2_R, P2Y_2_ receptor.

Finally, we asked whether the P2Y_2_R participates in adipogenesis *in vitro* and *in vivo*. For the *in vitro* study, we used an *Adipogenesis Assay Kit* to quantify the Oil Red-O staining of the formed lipid droplets as evidence of human white adipocyte adipogenesis. Figure 6, *A* and *B* show that cotreatment of the differentiating cells with AR-C118925 for 6 days dose dependently inhibited Oil Red-O staining with a maximal inhibition of about 35%. This data indicates that the P2Y_2_R is partially involved in human visceral adipocyte adipogenesis *in vitro*.

To evaluate the role of P2Y_2_R *in vivo*, we employed the P2Y_2_R-KO mice and its controlled WT mice. Figure 6*C* shows that at the 2-months age, both WT and KO mice had nearly identical weights; however, after feeding with a Western diet, the KO mice gained significantly less weight at the 2-, 3-, and 4-months time points compared to the WT mice. We further isolated the epididymal fat pods (eWAT), and Figure 6*D* shows that the KO mice had a significantly smaller eWAT than the WT mice. Figure 6*E* shows that compared with the healthy mice (WT fed with a chow diet), feeding the WT mice with the Western diet significantly increased the cell size of the adipocytes as expected (also see Fig. 6*F*). Surprisingly, we did not observe any change of the cell size from the KO mice as compared to the WT mice that all were fed with the same Western diet for 4 months (Fig. 6, *E* and *F*). On the other hand, we did observe an upregulation of P2Y_2_R expression in the eWAT of the WT mice fed with the Western diet compared to the healthy control mice (Fig. 6*E*). In addition, more macrophage staining on the eWAT was found in the WT mice fed with the Western diet compared to the healthy controls and that the KO mice showed much fewer macrophages than the WT fed with the same Western diet (Fig. 6*E*). Collectively, these data indicate that the P2Y_2_R is involved in mouse visceral adipogenesis *in vivo*.

## Discussion

In the present study, we show for the first time that the human P2Y_2_R is not only upregulated during terminal differentiation of visceral adipocytes but also is the only one among the eight known P2Y receptors that is further upregulated by inflammation. In addition, we have demonstrated that activation of the P2Y_2_R selectively impairs insulin receptor-mediated AKT signaling in mature human adipocytes without affecting the ERK1/2 pathway. Moreover, we identified that insulin-induced glucose uptake in mature adipocytes with the increase of PIP3 and the membrane translocation of GLUT4 is all reduced by P2Y_2_R activation. These findings provided a new mechanistic link between adipocyte P2Y_2_R and insulin resistance.

A few studies have reported that P2Y_2_R is related to obesity and insulin resistance. For example, Zhang *et al.* (25) explored the role of P2Y_2_R in high-fat diet–induced obesity *in vivo*. They fed WT and global P2Y_2_R KO mice with a high-fat diet and found that KO mice gain weight slower with improved glucose tolerance and insulin sensitivity than WT mice, and P2Y_2_R facilitated adipogenesis and inflammation. However, another study by Adamson S *et al.* (26) demonstrated that the P2Y_2_R on myeloid cells is essential in mediating acute inflammation but is dispensable for developing insulin resistance in diet-induced obese mice. They transplanted WT mice with either WT or P2Y_2_R-null bone marrows and treated them with endotoxin as a model of acute inflammation or fed a high-fat diet as a model of chronic inflammation. These studies showed seemingly conflicting results, which let us consider which tissue or cellular P2Y_2_R is responsible for the phenotype that protected mice from high-fat diet–induced obesity and insulin resistance. We, therefore, hypothesized that the adipocyte P2Y_2_R might be directly responsible for diet-induced obesity and insulin resistance. Using human visceral preadipocytes as a model, we found that during preadipocyte-to-mature adipocyte differentiation, P2Y_2_R was upregulated, and cotreatment with ARC-118925, a specific P2Y_2_R antagonist, inhibited adipogenesis. This result supports our notion that the adipocyte P2Y_2_R is involved in terminal adipogenesis, a new finding extended prior observations showing P2Y_2_R involvement in stem cell differentiation into preadipocytes (28). Since previous studies (25, 26) used a high-fat diet containing 60% calories as fat, we applied a Western diet (42% fat) in the current study to mimic our lifestyle better. We found that feeding with a Western diet, the P2Y_2_R KO mice gained significantly less weight over the 4 months than the WT mice, with less epididymal fat mass. This finding is also consistent with prior studies using the high-fat diet (25, 26), suggesting that the role of P2Y_2_R in obesity can be generalized to different animal models. Interestingly, although we found a smaller eWAT mass in KO mice, our histology results indicate that the size of the eWAT adipocytes was not different between the WT and KO mice. Since adipose tissue expands by a combination of an increase in adipocyte size (hypertrophy) and number (hyperplasia), it is highly possible that the P2Y_2_R may just be involved in adipocyte hyperplasia but not the hypertrophy process. This needs to be further confirmed in future studies and is better in an adipocyte-specific P2Y_2_R KO mouse model in which insulin resistance through glucose tolerance and insulin tolerance tests can be determined.

Adipogenesis involves the dynamic process of both lipogenesis and lipolysis. The current study found that stimulation of the P2Y_2_R with UTP, but not ATP, inhibited lipolysis, as evidenced by a decreased glycerol release after UTP but not ATP treatment. Others have observed this apparent agonistic discrepancy in the role of P2Y_2_R in rat adipocytes (29). However, it should be noted that ATP can activate the P2Y_11_ receptor, which is highly expressed in those mature human adipocytes based on our RT-PCR results, and that ATP degradation product of adenosine can activate adenosine receptors, both of which can increase intracellular cAMP levels, a master regulator of adipocyte lipolysis. Thus, it is plausible that UTP selectively activates the P2Y_2_R to inhibit lipolysis, but ATP simultaneously activates both the pro- and anti-lipolysis P2Y_11_ and P2Y_2_ receptors, respectively, in our system. Our finding is consistent with a recent report showing that UTP-activated P2Y_2_R inhibits basal lipolysis in human adipocytes (27, 29). They found that treatment of their cells with the P2Y_2_R antagonist increased intracellular cAMP levels, which they believed was due to P2Y_2_R-mediated upregulation of PDE enzyme activity. Since we could not confirm the regulatory role of P2Y_2_R on cellular PDE enzyme activity, we hypothesized that the P2Y_2_R might be coupled with the Gi proteins in addition to the traditional Gq proteins in our cells. Indeed, we found that stimulation of the P2Y_2_R with UTP dose-dependently increased GTP binding to the Gαi proteins in crude cell membranes. In addition, pretreatment of the cells with PTX, a specific inhibitor of Gi proteins, significantly blocked P2Y_2_R signaling. These results and other findings (27) support our notion that the adipocyte P2Y_2_R couples with the Gi protein to decrease intracellular cAMP, suppressing lipolysis. This notion was further supported by our new finding that activation of the P2Y_2_R by UTP inhibited forskolin-induced cellular cAMP elevation, which was fully prevented when the cells were pretreated with the P2Y_2_R antagonist AR-C 118925 or the Gi protein inhibitor PTX. To the best of our knowledge, this is the first time to propose a dual G protein coupling (Gi and Gq) mode for the P2Y_2_R. This is possible given the fact that there is a massive upregulation of the Gi proteins during terminal adipogenesis, as we found in this study, and other P2Y subtype receptor, such as the P2Y_11_, has been confirmed to couple with both Gs and Gq proteins.

It has been known that chronic inflammation mediated by macrophages and other immune cells in adipose tissue contributes to obesity and insulin resistance (17, 30, 31). The existing concept of macrophages releasing pro-inflammatory factors like TNFα to activate the adipocyte JNK signaling pathway that leads to insulin receptor desensitization by JNK phosphorylation of insulin receptor substrate is under debate (18). This concept cannot explain why some post-insulin receptor signaling pathways, such as ERK1/2, are not compromised as much as the AKT pathway, suggesting that an additional unknown mechanism can selectively impair the insulin receptor-induced AKT pathway. However, it has been well established that the PI3K/AKT pathway promotes GLUT4 translocation from cytosol to the cell membrane and the uptake of excess glucose from the bloodstream (32), which plays an essential role in developing obesity and type 2 diabetes (14). PI3K is a kinase capable of phosphorylating the PIP2 into PIP3, and the three-position phosphate group of PIP3 can bind to both PDK1 and AKT proteins and recruit AKT protein at the plasma membrane, leading to initial AKT activation (14). On the other hand, the P2Y_2_R was known to activate PLC *via* Gq proteins to mediate the production of inositol 1,4,5-triphosphates (IP3), leading to increased intracellular Ca^2+^. Since both PIP3 and IP3 formation require the presence of the same substrate PIP2, it is likely that competition between P2Y_2_R-induced IP3 generation and insulin-induced PIP3 production. Once the P2Y_2_R is upregulated and/or an overwhelming increase of extracellular ATP/UTP, the mature adipocytes may consume more PIP2 into IP3 instead of PIP3. We believe this is a more direct and neglected new mechanism underlying insulin resistance in inflamed adipocytes. Several lines of evidence support our notion: (1) We found that activation of the P2Y_2_R triggered an inhibitory effect on insulin-induced PIP3 production; (2) In mature adipocytes, activation of the P2Y_2_R inhibited insulin-induced AKT but not ERK1/2 phosphorylations; (3) Insulin-induced GLUT4 membrane translocation was dramatically inhibited by P2Y_2_R stimulation; (4) Insulin receptor-mediated glucose uptake was significantly suppressed by ATP/UTP activation of the P2Y_2_R; (5) TNFα-induced adipocyte inflammation selectively upregulated P2Y_2_R *in vitro*; (6) Western diet feeding of the mice induced more P2Y_2_R expression in visceral adipocytes *in vivo* compared with healthy control mice fed with chow diet; and (7) The minimal required dose of UTP for P2Y_2_R-mediated AKT inhibition was around 100 μM in non-inflamed mature adipocytes; however, it was drastically decreased to 0.1 μM in TNFα-primed adipocytes which had a much higher level of P2Y_2_R. Thus, we have provided compelling evidence indicating that the adipocyte P2Y_2_R can lead to insulin resistance by inhibiting the PI3K/AKT/GLUT4 pathway. This new finding may also explain some historic observations without a solid mechanism. For example, as early as 1974, Chang KJ & Cuatrecasas P first reported that extracellular ATP at 5 to 50 μM inhibited insulin-induced glucose uptake in isolated rat adipocytes (33). However, they postulated in that report that this might be due to an unknown biochemical phosphorylation event at the cell membrane surface where extracellular ATP acts as a phospho-donor (33). Four years later, another independent group confirmed the extracellular ATP inhibitory effect on insulin-induced glucose uptake in rat adipocytes (34). However, no proven mechanisms have been reported concerning how extracellular ATP suppresses insulin-induced glucose transport in adipocytes since then. We recently reported that extracellular ATP through P2Y_2_R activation in human hepatocytes blocks insulin signaling to the AKT pathway by decreasing PIP3 generation (35), consistent with our earlier findings in human skeletal muscle cells (36). Thus, it seems to be a general mechanism for P2Y_2_R control of insulin-AKT signaling in significant metabolic tissues or cells.

The unexpected findings of our Ca^2+^ mobilization assays in the current study triggered our interest that ATP/UTP stimulated no significant response in healthy preadipocytes and mature adipocytes. In contrast, there were substantial Ca^2+^ signals in response to P2Y_2_R activation in mature adipocytes primed with inflammation. It should be noted that in healthy mature adipocytes, the upregulated P2Y_2_R are still functional even though no Ca^2+^ signaling was detectable after adding exogenous ATP or UTP because, in those cells, the ERK1/2 and JNK pathways were found to be activated in response to P2Y_2_R activation. Thus, it is possible that in healthy mature adipocytes, the P2Y_2_R may be primarily coupled with Gi proteins, which were supported by our GTP binding assay and Western blotting assays showing that the Gi proteins were dramatically upregulated after adipocyte differentiation and that PTX treatment nearly abolished P2Y_2_R signaling to the ERK1/2 pathway and cAMP inhibition. Alternatively, the P2Y_2_R-Gq-Ca^2+^ pathway might be constitutively active and “saturated” due to the constitutive release of cellular ATP, as reported recently (27). Indeed, our finding that both P2Y_2_R-selective antagonist AR-C118925 and siRNA treatment significantly decreased basal intracellular Ca^2+^ levels support such a mode. On the other hand, the massive additional upregulation of the P2Y_2_R after TNFα treatment may render the spare P2Y_2_R the chance to couple with additional Gq proteins, leading to the observed Ca^2+^ signaling. Such a new dual G-protein coupling mode under inflammation for the adipocyte P2Y_2_R needs further investigation.

In summary, by using human visceral preadipocytes and a mouse model fed with a Western diet, we confirmed previous findings by others in different models that the P2Y_2_R is involved in terminal adipocyte differentiation *in vitro* and *in vivo*. In addition, we report the first evidence that activation of human adipocyte P2Y_2_R by ATP/UTP contributes to insulin resistance in mature adipocytes, in which a specific insulin receptors downstream signaling pathway, including PIP3 production, AKT phosphorylation, GLUT4 translocation, and glucose uptake is impaired by P2Y_2_R activation. Our finding suggests adipocyte P2Y_2_R is a potential new drug target in preventing and treating insulin resistance-related obesity and type 2 diabetes.

## Experimental procedures

### Cell culture, differentiation, and stimulation

Human Visceral Preadipocytes (preadipocytes) were purchased and cultured in a preadipocyte growth medium (ScienCell Research Laboratories) supplemented with 100 units/ml penicillin(Lonza), 100 μg/ml streptomycin (Lonza), and 10% heat-inactivated fetal bovine serum (FBS) (HyClone, Thermo Fisher Scientific) at 37 °C in a humidified atmosphere of 5% CO_2_. Preadipocytes were used between the third and eighth passages, seeded at 10^5^ cells/well in a six-well plate, and grown for 24 h, reaching ∼90 to 100% confluence. Preadipocytes can be further differentiated into mature adipocytes using a preadipocyte differentiation medium (ScienCell Research Laboratories). The purchased differentiation medium has the following main ingredients: 10% FBS, 10 μg/ml insulin, 0.5 mM 3-isobutyl-1-methylxanthine, and 1 μM dexamethasone (ScienCell Research Laboratories). For differentiation experiments, preadipocytes were plated in 6-well plates and grew until confluent, after which the cell medium was replaced by the above-mentioned differentiation medium, which was replaced every 2 days. The cells were differentiated for different times, as specified in the figures. For Western blotting assays of receptor signaling, the cells were differentiated for 6 days and then switched to a purchased mature adipocyte maintenance medium containing 10% FBS, 10 μg/ml insulin, and 1 μM dexamethasone (ScienCell Research Laboratories). Mature adipocytes were continuously maintained in the adipocyte maintenance medium. Before stimulation, cells were serum-starved for 24 h in the basal adipocyte medium without any differentiation cocktail supplement. Whenever inhibitors were used, cells were pretreated with the inhibitors for 45 min before cell stimulation. 3T3-L1 cells from ATCC were cultured in regular DMEM medium with 10%FBS, and the cells were differentiated into mature mouse adipocytes using the same differentiation medium as in the above human cells.

### RT-PCR and real-time PCR analysis

Total RNA and DNA were extracted from pre- and mature adipocytes according to the manufacturer’s protocol for the RNeasy and DNeasy kits (Qiagen). On-column DNA digestion was carried out during RNA extraction. For synthesizing the first strand of cDNA, 1 μg of total RNA after DNase (Ambion) treatment was reverse-transcribed using a cDNA synthesis kit (Applied Biosystems). The cDNA samples were then amplified by general PCR or Real-time PCR using 2.5 units of Taq DNA polymerase (Qiagen) and SYBR Green reagents (Applied Biosystems), respectively. The sequences of primers for human receptors are as follows: P2Y_1_R, forward: 5′ - TGG CGG GAG ATA CTT TCA - 3′, reverse: 5′- GGA GAT TCT TGT GCC TTC AC - 3′; P2Y_2_R (240 bp), forward: 5′ - CCT CAA GAC CTG GAA TGC GT - 3′, reverse: 5′ - TGG AAT GGC AGG AAG CAG AG - 3′; P2Y_2_R (600 bp), forward: 5′ - GTG CTC TAC TTC CTG GCT - 3′, reverse: 5′ – CTG AAG TGT TCT GCT CCT AC - 3′; P2Y_4_R, forward: 5′ - TCT ATA AAG TGA CTC GGC CC - 3′, reverse: 5′ - GGC TTC CCG TGT TAC AAT - 3′; P2Y_6_R, forward: 5′ - TGG GCA GCC ATG GAA T - 3′, reverse: 5′ - GAG CAA GGT TTA GGG TGT AC - 3′; P2Y_11_R, forward: 5′ - GCG GCC TAC AGA GCG TAT AG - 3′, reverse: 5′ - CTG GGG CTC TGA CGG TTT AG - 3′; P2Y_12_R, forward: 5′ - CCA AAC TGG GAA CAG GAC CA - 3′, reverse: 5′ - AGG GTG TAA GGA ATT CGG GC - 3′; P2Y_13_R, forward: 5′ - GGT GAC ACT GGA AGC AAT - 3′, reverse: 5′ - ACC CAC AGA GCC AAA GTA - 3′; P2Y_14_R, forward: 5′ – CTC ATT ACA GCT GCC AGT - 3′, reverse: 5′ – TTG GAA GAG GGT AGG AAC TC - 3′. The sequences of primers for mouse receptors are as follows: P2Y_1_R, forward: 5′ – GAG GTG CCT TGG TCG GTT G -3′, reverse: 5′- CGG CAG GTA GTA GAA CTG GAA - 3′; P2Y_2_R, forward: 5′ – CTG GAA CCC TGG AAT AGC ACC - 3′, reverse: 5′ – CAC ACC ACG CCA TAG GAC A - 3′; P2Y_4_R, forward: 5′ – ATG ACC AGT GCA GAC TCC TTG - 3′, reverse: 5′ – GAG GCA ACA GGA TGA ACT TGA - 3′; P2Y_6_R, forward: 5′– GTG AGG ATT TCA AGC GAC TGC - 3′, reverse: 5′ – TCC CCT CTG GCG TAG TTA TAG A - 3′; P2Y_12_R, forward: 5′ – CCC TGT GCG TCA GAG ACT AC - 3′, reverse: 5′ – CAA GCT GTT CGT GAT GAG CC - 3′; P2Y_13_R, forward: 5′ – ATG CTC GGG ACA ATC AAC ACC - 3′, reverse: 5′ – GAT GTG GAC GAA CAC CCA GAG - 3′; P2Y_14_R, forward: 5′ – TGG CAC AAG GCG TCT AAC TAT - 3′, reverse: 5′ – GAC TTC CTC TTG ACG GAG GTG - 3′. The sequences of primers for adipogenic markers are as follows: C/EBPα, forward: 5′– TAT AGG CTG GGC TTC CCC TT - 3′, reverse: 5′- AGC TTT CTG GTG TGA CTC GG - 3′; C/EBPβ, forward: 5′- GGG AGC CCG TCG GTA ATT TT- 3′, reverse: 5′- CAT GTG CGG TTG GTT TGG AC- 3′; PPARγ, forward: 5′- ACC CAG AAA GCG ATT CCT TCA - 3′, reverse: 5′– CAC GGA GCT GAT CCC AAA GT - 3′; GAPDH, forward: 5′- CGA CCA CTT TGT CAA GCT CA - 3′, reverse: 5′– AGG GGA GAT TCA GTG TGG TG - 3′. The general PCR condition was for 40 cycles of the following: jump start for 2 min at 95 °C, denaturation for 1 min at 95 °C, annealing for 1 min at 60 °C, and extension at 72 °C for 1 min. The resulting PCR products were resolved on a 1.5% agarose ethidium bromide gel, and the bands were visualized with ultraviolet light.

The Real-time PCR mixture (20 μl) contained 0.5 μm concentration of each primer, 4 μl of water, 10 μl of SYBR Green mixture, and 5 μl of cDNA. The samples were placed and sealed in 96-well plates with the following reaction conditions: initial PCR activation step (5 min at 95 °C) and cycling steps (denaturation for 1 min at 95 °C, annealing for 1 min at 60 °C, extension for 2 min at 72 °C; 40 cycles). An internal control, GAPDH, was amplified in separate wells. The threshold cycle (Ct) value and the efficiency of PCR amplification for each set of primers were determined using the accompanying software. We used the comparative cycle threshold ΔΔCt method for relative quantification of gene expression.

### Western Blotting assay

After stimulation for indicated times, cells were lysed, and standard Western blotting was performed as previously described (37). The individual primary antibodies used were anti-p-AKT, anti-p-ERK1/2, anti-p-p38, anti-p-JNK, and anti-GLUT4 (Cell Signaling). Equal protein loading was verified by stripping off the original antibodies and re-probing the membranes with the primary antibody anti-GAPDH, anti-β-Actin, anti-β-tubulin, anti-Caveolin-, or anti-total AKT, ERK1/2, p38, or JNK (Cell Signaling).

### Calcium mobilization assay

Intracellular Ca^2+^ concentration was measured using the FluoForteTM Calcium Assay kit (Enzo Life Sciences). Pre and mature adipocytes were plated in 96-well plates at 4 × 10^4^ cells/100 μl/well. After starvation, 100 μl of Dye-loading solution was added. The cells were further incubated for 45 min at 37 °C and 15 min at room temperature before stimulation. The receptor-mediated Ca^2+^ mobilization was determined as previously described (38). In the antagonist inhibition experiment, cells were pretreated with the antagonist for 45 min before agonist addition. A time-response curve of intracellular Ca^2+^ signal was recorded with a fluorometer plate reader (BMG FLUOstar) with a 490/525 nm bandpass filter, the results of which were shown as relative fluorescence units.

### Glucose uptake assay

Measuring the glucose uptake was performed by using Glucose Uptake-Glo Assay (Promega). Briefly, preadipocytes were plated in a preadipocyte growth medium in a 6-wells plate. Once preadipocytes reach 100% confluence, replace the preadipocyte growth medium with the preadipocyte differentiation medium and change with the fresh medium every 2 days until fully differentiated. Mature adipocytes were starved overnight and replaced with the glucose-free medium the next day, 3 h before stimulation. Mature adipocytes were stimulated by insulin 10 nM, ATP/UTP 100 μM, and insulin 10 nM + ATP/UTP 100 μM for 40 min at 37 °C in a humidified atmosphere with 5% CO_2_. For P2Y_2_R inhibition, the cells were pretreated with 3 μM AR-C 118925 for 45 min before insulin and ATP/UTP stimulation. After that, mature adipocytes were incubated with 100 μM 2-NBDG, a fluorescently tagged glucose analog, for 20 min at room temperature. At the end of the treatment, the plates were centrifuged for 5 min at 500 rpm at room temperature, and the supernatant was aspirated and replaced with 200 μl of cell-based assay buffer. Then, the plates were centrifuged again, and the supernatant was aspirated with 100 μl of 0.1 M HCL and shaken at 250 rpm for 30 min. Transfer the supernatant to a new plate and read with Glomax 96 Microplate Luminometer (excitation/emission = 485/535 nm, Promega).

### In-cell PIP3 Elisa assay

Intracellular PIP3 levels were evaluated as we previously reported (35). Briefly, preadipocytes were seeded in the black 96-well plate with clear bottom and differentiated into mature adipocytes. After starvation for 24 h and replaced with the fresh starvation medium for 3 h, mature adipocytes were stimulated by insulin with or without ATP/UTP for 5 min. Cells were fixed with fixation buffer 100 μl for 15 min at room temperature after removing the starvation medium. Cells were rinsed three times with a pre-chilled wash buffer of 200 μl. Buffer 1 plus 100 μl was applied to block and permeabilize the cells for 45 min on ice. The primary anti-PIP3 antibody was diluted in buffer 2. Cells were then incubated with the primary antibody solution 100 μl for 60 min on ice. Cells were rinsed twice with buffer 1100 μl for 5 min and four times with 1× wash solution 300 μl. Hundred microliters of streptavidin solution was added and incubated for 45 min at room temperature with gentle shaking. The streptavidin solution was discarded and washed four times with 1× wash solution 300 μl. TMB One-Step substrate Reagent 100 μl was added to each well and incubated for 30 min at room temperature in the dark with gentle shaking. Stop solution 50 μl was added to each well. The plate was read at 450 nm immediately by SpectraMax iD3 (Molecular Devices) *via* SoftMax Pro 7.1 software.

### Silencing of P2Y_2_R by siRNA

To knock down the P2YR, mature adipocytes were transfected with the four-sequence pool (ON-TARGET plus SMART pool L-003688-00-0005, human P2RY2, NM-002564, Dharmacon) using DharmaFECT 4 Transfection reagent following the manufacturer’s protocol. Briefly, preadipocytes were seeded in 6-well plates at 100% confluence and differentiated for 6 days; the differentiation medium was replaced with adipocyte growth medium before transfection. DharmaFECT 4 and siRNA products were incubated separately in the starvation medium at room temperature for 5 min. Mixtures were combined, incubated for another 20 min, and added to cells at a final concentration of 5 μl/ml DharmaFECT 4 and 25 nm siRNAs. Cells in the control group were treated with DharmaFECT 4 plus a scrambled control siRNA. The real-time PCR assay was performed to confirm the decrease of P2Y_2_R mRNA after 48 h post-transfection. Calcium release assay and Western blotting were employed to verify knock-down efficiency 72 h post-transfection.

### Membrane protein isolation

The Mem-PER Plus Membrane Protein Extraction Kit (Thermo Fisher Scientific) was applied to isolate membrane protein. Preadipocytes were seeded in the 6-well plate and differentiated into mature adipocytes. After starvation, mature adipocytes were stimulated by insulin with or without ATP/UTP for 10 min. Mature adipocytes were resuspended by scraping the cells off the surface of the plate with a cell scraper and then centrifuged at 300*g* for 5 min. The cell pellets were washed with 3 ml of Cell Wash Solution and centrifuged at 300*g* for 5 min. After the supernatant was removed, cells were suspended in 1.5 ml of Cell Wash Solution and transferred to a 2 ml centrifuge tube and centrifuged at 300*g* for 5 min, then discarded the supernatant. Permeabilization buffer 0.75 ml was added to the cell pellet and vortexed briefly, then incubated for 10 min at 4 °C with constant mixing. Permeabilized cells were centrifuged at 16,000*g* for 15 min, then discarded the supernatant. Solubilization buffer 0.5 ml was added to the cell pellet and resuspended by pipetting, then incubated for 30 min at 4 °C with constant mixing. The tube was centrifuged at 16,000*g* for 15 min at 4 °C, and the supernatant was transferred to a new tube. Lysis buffer was added to the supernatant and then boiled for 5 min. The sample lysates were stored at −20 °C for future use.

### Adipogenesis assay

The Adipogenesis assay kit (Cayman Chemical) was used to study the role of P2Y_2_R in adipogenesis. Preadipocytes were seeded in a 96-well plate and differentiated to mature adipocytes. P2Y_2_R antagonist was added together during differentiation to inhibit P2Y_2_R activity. Seventy five microliters of Lipid Droplets Assay Fixative was added to each well and incubated for 15 min. Wells were washed with 100 μl of wash solution twice for 5 min each. Seventy five microliters of Oil-Red O working solution was added to all wells, including the background wells containing no cells, and incubated for 20 min after the wells were dried completely. Oil Red O solution was removed, and cells were washed with distilled water several times until the water contained no visible pink color. The wells were washed with 100 μl of wash solution twice for 5 min each. After the wells dried completely, 100 μl of dye extraction solution was added to each well and mixed for 30 min. The absorbance was read at 490 to 520 nm with a 96-well plate reader (SpectraMax iD3, Molecular Devices).

### Adipolysis assay

The EnzyChrom Adipolysis Assay Kit (BioAssay Systems) was used to measure glycerol released during adipolysis and explore the effect of insulin and P2Y_2_R activation on adipolysis. Preadipocytes were seeded in a 6-well plate and then differentiated with the preadipocyte differentiation medium until fully differentiated. After starvation, mature adipocytes were stimulated by UTP, isoproterenol, or both. The cell culture supernatants were collected every 1 h and stored at −80 °C immediately. A 100 μg/ml glycerol standard was prepared and further diluted into 60 μg/ml and 30 μg/ml with a starvation medium to establish the standard curve. Ten microliters of culture medium and glycerol standard were transferred to a 96-well plate in separate wells and mixed with 100 μl working reagent (100 μl assay buffer, 2 μl enzyme mix, 1 μl ATP, and 1 μl dye reagent mixing). After incubation for 20 min at room temperature, the plate was read at 570 nm by a plate reader (SpectraMax iD3, Molecular Devices).

### Gi-protein binding assay

The GTP Gi binding assay kit (PerkinElmer, cisbio) with HTRF detection was used to detect the Gi protein activation in the membrane following agonist stimulation. Preadipocytes were seeded in a 6-well plate and then differentiated with the preadipocyte differentiation medium until fully differentiated. Mature adipocytes were washed 3 times with PBS, then scraped and harvested by a rubber policeman into a total volume of 30 ml of 25 mmol/l sodium phosphate (pH 7.4) and 5 mmol/l MgCl_2_. The suspended cells were lysed by 30-s homogenization and 10-s sonication on ice. The cell lysates were centrifuged at 30,000*g* for 15 min at 4 °C. The pellet was resuspended and centrifuged again. The resulting crude cell membrane was suspended in an appropriate volume of the stimulation buffer (stimulation buffer #3 supplemented with 0.5 μM of GDP and 50 mM of MgCl_2_). Twenty microliters of working mix (5 μl of stimulation buffer, 5 μl of 4× agonist, 5 μl of 4× detection reagent mix, and 5 μl of crude cell membrane) and twenty microliters of positive control (10 μl of stimulation buffer #3, 5 μl of Gi protein control, and 5 μl of 4× detection reagent mix) were added into a half-volume white 96-well plate and incubated overnight at 37 °C. The fluorescence ratio (665 nm/620 nm) was read by a plate reader (SpectraMax iD3, Molecular Devices).

### Cellular cAMP measurement

Intracellular cAMP levels were determined by using the Direct cAMP Elisa kit (Enzo Life Sciences) as the user manual instructed. Mature adipocytes in 12-well plates were starved overnight in a serum-free medium. Cells were pretreated with PTX (100 ng/ml, overnight) to inhibit Gi proteins or pretreated with AR-C 118924 (3 μM, 45 min) to block the P2Y_2_R, and Gi protein-mediated cAMP suppression was achieved by stimulation of the P2Y_2_R by UTP (100 μM) in the presence of forskolin (10 μM). During the treatment, 10 μM Rolipram was used to inhibit cellular phosphodiesterase activity. After 5 min, the incubation was terminated by pouring off the medium and adding 300 μl HCl (0.1 M). After cell lysis in the 37 °C incubator, the samples were centrifuged (1000*g* × 5 min), and the supernatants were acetylated as instructed by the kit. Hundred microliters of the acetylated supernatant was used for the Elisa assay, followed by the kit's protocol. cAMP levels were normalized for the protein content of each sample as determined by the Bradford assay.

### Animal handling

All animal procedures were reviewed and approved by the Auburn University Institutional Animal Care and Use Committee. 10-weeks-old P2Y_2_R global KO and WT C57bl/6 mice were purchased from Jackson Laboratories. All animals were housed on a 12 h light with a 12 h dark cycle and provided standard laboratory chow and water ad libitum. Male mice 8 weeks old were fed with a control chow diet or a Western diet from Envigo with catalog #TD.88137 (% KCALfrom: protein, 15.2%; carbohydrate, 42.7%; Fat, 42%) for at least 16 weeks for the experiment, and the body weights were measured every month. eWAT were isolated from euthanized mice and weighed before being fixed in 10% formalin in PBS overnight.

### Immunohistochemistry

Mouse eWAT fat slides were stained using the Superplus High Sensitive and Rapid Immunohistochemical Kit (Elabscience). Mouse fat slides were placed in Dewaxing/Antigen Retrieval working solution for 30 min to repair the antigen. Slides were blocked with peroxidase blocking buffer at room temperature for 15 min to eliminate endogenous peroxidase activity, then washed with PBS three times for 2 min. The primary antibody with a 1:400 dilution using antibody dilution buffer was added to the slides and incubated at 37 °C for 1 h, then washed with PBS three times for 2 min. A drop of Poly-peroxidase-anti-Rabbit/Mouse IgG was added to the slides and incubated at 37 °C for 30 min, then washed with PBS three times for 2 min. A drop of coloration solution was added to the slides, and color of tan or brownish-yellow appeared. Slides were washed with deionized water to terminate the chromogenic reaction. The hematoxylin stain was applied for a better image. The mounting liquid was applied to cover the tissue sections, and then cover glasses were used to cover the slides. H&E-stained eWAT sections were analyzed using the Adiposoft software from ImageJ to evaluate adipocyte size. At least five slides per mouse were analyzed, and the calculated mean of every mouse was used for further analysis.

### Materials

Human visceral preadipocytes and the growth and differentiation medium were purchased from ScienCell Research Laboratories. DNA primers were purchased from Integrated DNA Technologies. Recombinant human TNFα was purchased from R&D Systems. Purified ATP and UTP, Wortmannin, Pertussis Toxin, and A23187 were obtained from SIGMA-ALDRICH. P2Y_2_R antagonist AR-C 118925 was purchased from Tocris. P2Y_2_R and F4/80 antibodies were purchased from ABclonal. Other antibodies were purchased from Cell Signaling.

### Data analysis

Data are expressed as the mean ± SEM. The means of two groups were compared using Student’s *t* test (unpaired, two-tailed), and a one-way analysis of variance was used for the comparison of more than two groups with *p* < 0.05 considered to be statistically significant. Unless otherwise indicated, all experiments were repeated at least three times. Statistical analysis was performed with GraphPad Prism 9.0.

## Data availability

All data are contained within the article and supporting information.

## Supporting information

This article contains supporting information.

## Conflict of interest

The authors declare that they have no conflicts of interest with the contents of this article.
