# Supplementary material for: The P2Y2 receptor mediates terminal adipocyte differentiation and insulin resistance: Evidence for a dual G-protein coupling mode
Source: J Biol Chem. 2023 Dec 21;300(2):105589. doi: 10.1016/j.jbc.2023.105589 (PMC10828443; doi:10.1016/j.jbc.2023.105589)
Supplement: Supporting Figures S1–S3 [file mmc1.pdf]

**The P2Y2 Receptor Mediates Terminal Adipocyte Differentiation and Insulin Resistance: Evidence for a Dual G-protein Coupling Mode**

*Shenqi Qian, Yi Shi, Jared Senfeld, Qianman Peng, Jianzhong Shen**

**(Supplemental Figure 1, Figure 2, and Figure 3)**

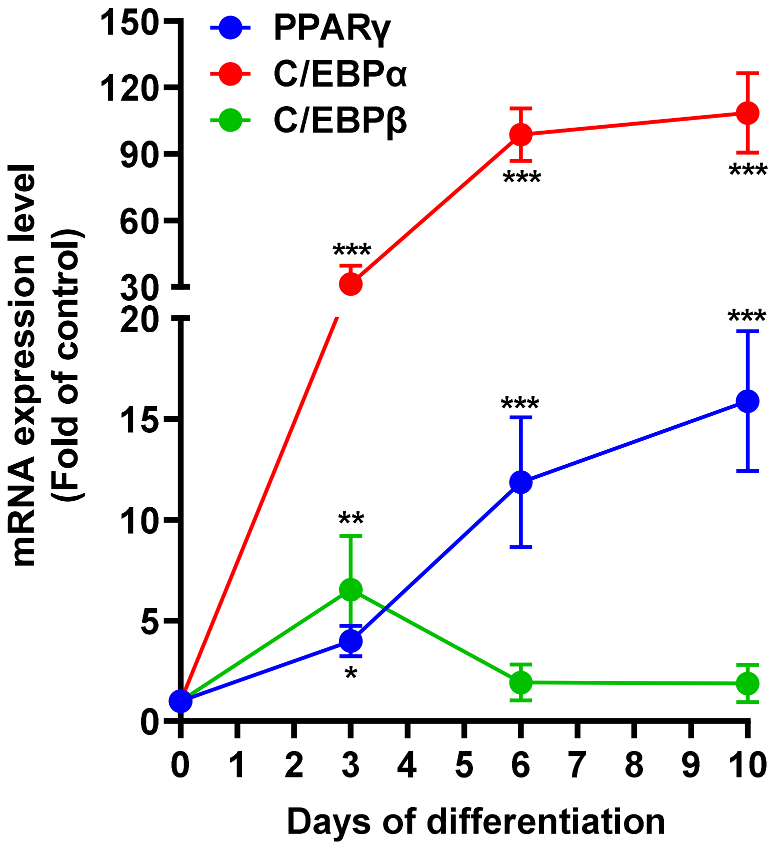


**Figure S1. mRNA expression changes of adipogenesis markers during human adipocyte differentiation.** The mRNA levels of PPARγ, C/EBPα, and C/EBPβ were determined by Real-time RT-PCR using 1.0μg total cellular RNAs purified from cultured human visceral preadipocytes that were differentiated for indicated days. *, $p<0.05$; **, $p<0.01$; ***, $p<0.001$; $n=3$.

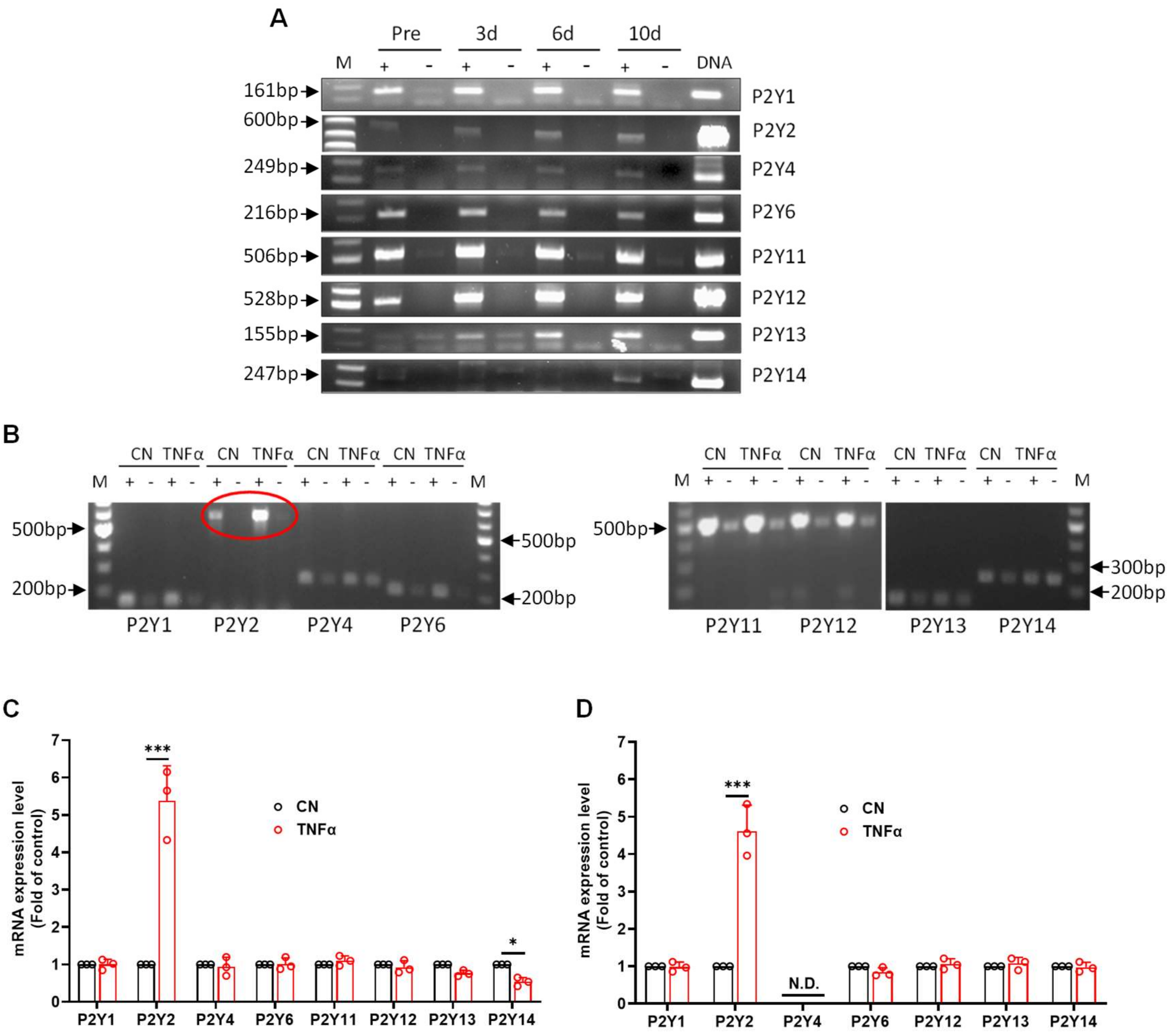


**Figure S2. Profile of gene expression changes of all P2Y receptors during terminal adipogenesis and inflammation.** The mRNA levels of eight known human P2Y receptors were determined by RT-PCR using 1.0μg total cellular RNAs purified from cultured human visceral preadipocytes that were differentiated for indicated days. Representative data from three independent experiments. For comparison purposes, the P2Y2 receptor data was reused from Figure 1C (**A**). Stimulation of the fully differentiated mature adipocytes by TNFα (10ng/ml) for 24 hours further selectively increased P2Y2R but not other subtypes' mRNA expression (highlighted in the red circle). Shown are representative of three independent RT-PCR experiments (**B**). Real-time quantitative RT-PCR analysis of all known eight human P2Y receptors' (**C**) and seven mouse P2Y receptors' (**D**) mRNA expression change in response to TNFα (10ng/ml) stimulation for 24 hours in the fully differentiated human mature adipocytes (**C**) and 3T3-L1 mouse adipocytes (**D**). *, $p<0.05$, ***, $p<0.001$; N.D. stands for non-detectable.

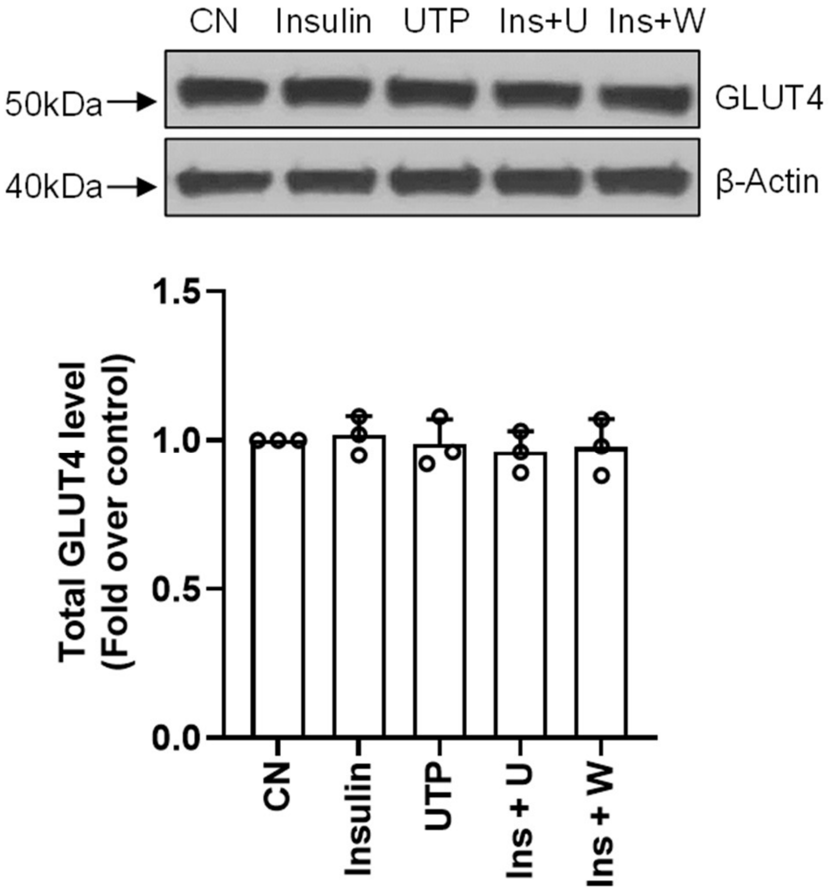


**Figure S3. No effect of P2Y2R activation on total cellular Glut4 expression in mature human adipocytes.** Total Glut4 protein expression was determined by Western blotting assay using total cellular lysates isolated from cultured mature human adipocytes stimulated with 10nM insulin with or without 100µM UTP co-treatment. 100nM Wortmannin was used as a control. β-Actin served as a protein loading control. Shown are representative of three independent experiments. No statistical difference was found between any of the two groups.
